# Nationwide hospitalization rates for salmonellosis in Chile, 2021–2024: A retrospective study

**DOI:** 10.1016/j.pmedr.2026.103462

**Published:** 2026-04-02

**Authors:** Constanza Pamela Rojas Navia, Rocío Belén Herrera Caracciolo, Ankiza Nicole Knezevic Altamirano, Sofía Ignacia Macari Jorquera, Juan Sanchis-Gimeno, Jessica Paola Loaiza Giraldo, Jose E. León Rojas, Juan José Valenzuela Fuenzalida

**Affiliations:** a Facultad de Medicina, Universidad Andrés Bello, Santiago 8370146, Chile; b Facultad de Ciencias de la Salud, Universidad Central del Valle del Cauca (UCEVA), Tuluá 763022, Valle del Cauca, Colombia; c GIAVAL Research Group, Department of Anatomy and Human Embryology, Faculty of Medicine, University of Valencia, 46001, Valencia, Spain; d Grupo de Investigación Bienestar, Salud y Sociedad, Escuela de Psicologia y Educación, Universidad de Las Américas, Quito 170124, Ecuador; e Departamento de Morfología, Facultad de Medicina, Universidad Andrés Bello, Santiago 8370146, Chile; f Departamento de Ciencias Química y Biológicas, Facultad de Ciencias de la Salud, Universidad Bernardo O'Higgins, Santiago 8370993, Chile

**Keywords:** Salmonellosis, Non-typhoidal *Salmonella*, Hospitalization rate, Epidemiology, Chile

## Abstract

Salmonellosis is a major enteric infection and a leading cause of diarrheal disease worldwide. In Chile, however, contemporary evidence on its hospital burden remains limited. This study aimed to estimate the hospitalization rate due to salmonellosis in Chile between 2021 and 2024 and to describe variations by sex, age group, and length of hospital stay.

**Methods:**

A retrospective, cross-sectional descriptive study was conducted using secondary data from the Department of Health Statistics and Information (DEIS) of the Chilean Ministry of Health and population estimates from the National Institute of Statistics (INE). Annual hospitalization rates were calculated per 100,000 inhabitants and stratified by sex and age group. Data were processed using Microsoft Excel.

**Results:**

Between 2021 and 2024, a total of 2672 hospital discharges due to salmonellosis were reported, corresponding to an overall hospitalization rate of 3.61 per 100,000 inhabitants. The lowest hospitalization rate was observed in 2022 (3.25), while the highest occurred in 2024 (4.29). A slight male predominance was observed during the study period (3.67 in men vs. 3.56 in women). Children under 5 years exhibited the highest hospitalization rate (11.74)**,** whereas adolescents aged 15–19 years had the lowest hospitalization rate (2.36). The mean length of hospital stay during the study period was 4.69 days.

**Conclusions:**

The hospitalization rate of salmonellosis remained relatively stable during the study period, suggesting a persistent baseline level of hospital-treated disease in Chile. Continued epidemiological surveillance is essential to monitor potential changes in hospitalization trends and guide public health strategies. The higher hospitalization rates observed in children under 5 years and older adults highlight vulnerable age groups that may require focused monitoring and healthcare planning.

## Highlights

• National hospital burden estimates of salmonellosis in Chile (2021–2024).
• Highest hospitalization rates in children under 5 and older adults.
• Post-pandemic increase in salmonellosis-related hospital discharges.
• Stable hospital length of stay during the study period.
• Findings support stronger surveillance and food safety policies.

## 1 Introduction

*Salmonella* enteriti*s* or salmonellosis is the name given to acute intestinal infection caused by infection with the bacterium *Salmonella*, a facultative Gram-negative bacillus capable of infecting multiple hosts and various cell types (Rosso et al., 2023). There are more than 2600 serotypes; grouped according to their somatic (O) and flagellar (H) antigens; allowing classification into typhoidal *Salmonella*; which typically cause systemic enteric fever; and non-typhoidal *Salmonella* (NTS)**;** which most commonly produce self-limited gastroenteritis. However; certain NTS strains can also cause invasive disease**;** particularly in immunocompromised individuals; children; and older adults. Regarding typhoid and non-typhoid *Salmonella*; it has been shown that among the first group; the Typhi O:9H:d serotype is recognized as the main cause of more than 11–20 million illnesses and approximately 210;000 deaths per year. However; together with serotypes Paratyphi A; B tartrate-negative; and C; they account for more than 22 million illnesses and approximately 250;000 deaths per year (Rosso et al., 2023; Galán, 2021).

In contrast, non-typhoid serotypes such as *Salmonella Enteritidis* and *Salmonella Typhimurium* are more frequently associated with self-limiting gastroenteritis. Of these, *S. enteritidis* is the most common strain in Latin America, accounting for 31% of cases, while *S. typhimurium* predominates in North America, accounting for 21% of cases (Muleta and Meseret, 2025; Alfaro-Mora, 2019). No specific serotype information was available for the years 2021–2024 in the databases used.

Infection occurs after ingestion of food or environmental sources contaminated with *Salmonella.* (mainly chicken meat, eggs, and dairy products). After oral acquisition, *Salmonella* moves through the intestinal tract until it reaches the large intestine, colonizing enterocytes where most replication occurs, triggering inflammation, invasion of the intestinal mucosa, and an imbalance in the host's microbiota. This causes an alteration limited to the intestine; however, in some cases, the infection can spread and cause systemic involvement (Muleta and Meseret, 2025). Systemic involvement should be considered particularly relevant in immunocompromised patients, since humans can suffer chronic colonization in the biliary system, cecum, and urinary tract when they have morphological alterations due to malformations or lithiasis.

The most common clinical presentation includes gastroenteric syndrome, where symptoms are diarrhea, fever, abdominal pain, and vomiting, which may also be accompanied by laboratory alterations such as leukocytosis, thrombocytosis, anemia, and increased CRP. The diagnosis is mainly clinical; however, laboratory techniques such as molecular tests or stool culture can also be used (Parra-Payano et al., 2019).

Since most cases are self-limiting, only symptomatic treatment is usually provided. However, if the disease progresses, the use of antibiotics may also be useful, as may hospitalization, which is generally indicated in immunosuppressed patients, those at extreme ages (infants, preschoolers, and older adults), or in cases of infection with more invasive serotypes (Mayo Clinic, 2025; Harrison, 2021). Hospitalization may also be necessary when complications such as neurological impairment, hepatitis, intestinal perforation, sepsis, shock, or disseminated intravascular coagulation occur. In such cases, admission may even require referral to an intensive care unit (Fattinger et al., 2021).

Salmonellosis is an important foodborne infection worldwide and a recognized cause of diarrheal disease. Although most infections are self-limited, severe cases may require hospitalization, particularly among vulnerable populations such as young children, older adults, and immunocompromised individuals. In Chile, contemporary epidemiological data on the hospital burden of salmonellosis remain limited, particularly regarding trends by age, sex, and severity indicators such as length of hospital stay. This lack of updated national evidence may hinder adequate surveillance and health system planning. Therefore, the aim of this study was to estimate to estimate the hospitalization rate due to salmonellosis in Chile between 2021 and 2024 and to analyze variations according to sex, age group, and length of hospital stay.

## 2 Methods

### 2.1 Study design and population

A retrospective, observational, and descriptive study was conducted to estimate the hospitalization rate due to salmonelosis Chile between 2021 and 2024. The analysis was carried out at the national level using annual data corresponding to the study period.

### 2.2 Measures

Data on hospital discharges were obtained from the Department of Health Statistics and Information (DEIS) of the Chilean Ministry of Health. Population data required for incidence calculations were obtained from the National Institute of Statistics (INE), including population estimates from the 2024 Census. The study included hospital discharges due to salmonellosis stratified by sex, age group, and length of hospital stay. For reference, the DEIS database reported 643 patients in 2021 (302 men and 341 women), 600 patients in 2022 (300 men and 300 women), 636 patients in 2023 (334 men and 302 women), and 793 patients in 2024 (380 men and 413 women).

### 2.3 Statistical analysis

Descriptive statistics were used to summarize hospital discharge data. Annual hospitalization rates were estimated per 100,000 inhabitants by year, sex, and age group using the following formula: Hospitalization rate = (Number of hospital discharges due to salmonellosis in a given year / Total population at risk) × 100,000. Because the database captures hospital discharge records rather than incident infections in the population, the results are interpreted as hospitalization rates rather than true disease incidence. Data were processed and analyzed, and results were organized into tables and graphical representations. Because the study used aggregated national administrative data across a limited number of years, the analysis was primarily descriptive and no formal statistical hypothesis testing was performed.

All analyses were performed using Microsoft Excel (Microsoft Corporation, Redmond, WA, USA).

### 2.4 Ethical considerations

This study was conducted using anonymized secondary data obtained from publicly available national databases. According to local regulations and institutional policies, ethical approval was not required. The corresponding author confirms that all procedures complied with applicable guidelines for the protection of human subjects.

## 3 Results

**Fig. 1:**
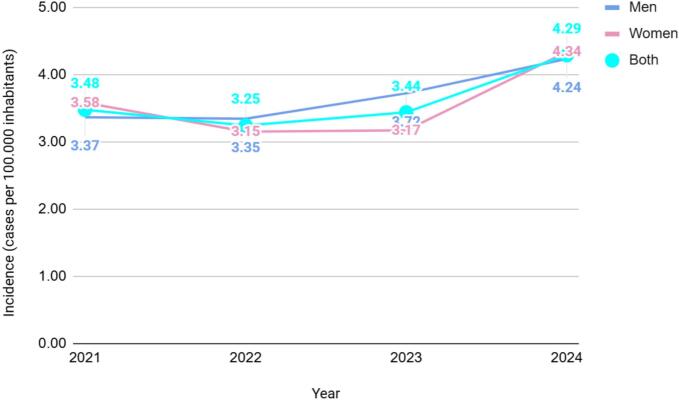
Hospitalization rate of salmonellosis (cases per 100,000 inhabitants) by sex in Chile, 2021–2024. In 2021 and 2024, hospitalization rates were slightly higher among women (3.58 and 4.34 cases per 100,000 inhabitants, respectively), whereas in 2022 and 2023 rates were higher among men (3.35 and 3.72, respectively). The largest sex difference occurred in 2023, when the hospitalization rate reached 3.72 cases per 100,000 inhabitants in men and 3.17 in women. Across the study period, the mean hospitalization rate was 3.67 cases per 100,000 inhabitants in men and 3.56 in women.

**Fig. 2:**
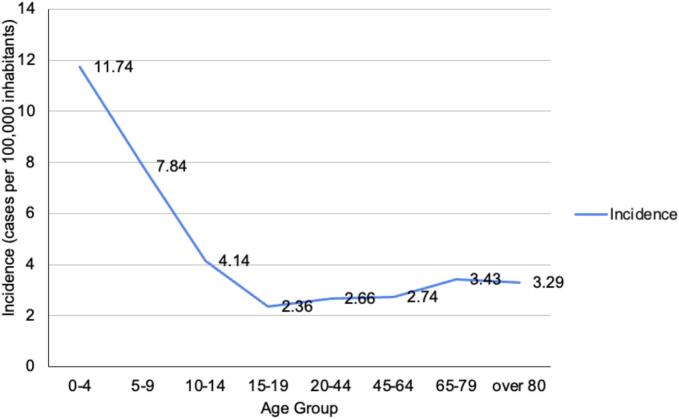
**Hospitalization rate of salmonellosis (cases per 100,000 inhabitants) by age group in Chile, 2021–2024.** The highest hospitalization rate occurred in children aged 0–4 years (11.74 cases per 100,000 inhabitants). Rates declined progressively with increasing age, reaching a minimum in adolescents aged 15–19 years (2.36 cases per 100,000 inhabitants). A slight increase was observed among older adults, with hospitalization rates of 3.43 and 3.29 cases per 100,000 inhabitants in the 65–79 and ≥ 80-year age groups, respectively. Overall, hospitalization rates were higher at the extremes of age.

**Fig. 3:**
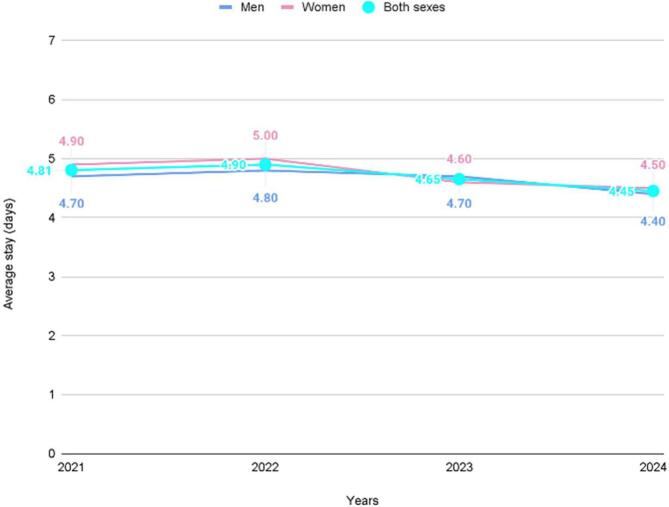
**Average length of hospital stay for salmonellosis (days) by sex in Chile, 2021–2024.** In the period studied between 2021 and 2024, the average hospital stay was determined to be 4.69 days, reaching its highest average in 2022 with a value of 4.90 days. However, from that year onwards, there was a progressive decrease in the length of stays, which continued until reaching a minimum of 4.45 days in 2024. According to the average length of hospital stay by gender, it was found that women had longer hospitalizations with an average of 4.73 days, showing a slight predominance compared to men, who had an average hospital stay of 4.64 days (Fig. 3).

A total of 2672 hospital discharges due to salmonellosis were recorded in Chile between 2021 and 2024, corresponding to an overall hospitalization rate of 3.61 cases per 100,000 inhabitants for the study period. Annual hospitalization rates per 100,000 inhabitants were 3.48 in 2021, 3.25 in 2022, 3.44 in 2023, and 4.29 in 2024, with the lowest value observed in 2022 and the highest in 2024. When stratified by sex, a slight male predominance was observed in the average hospitalization rate for the period (3.67 in men vs. 3.56 in women). In 2021 and 2024, the hospitalization rate was slightly higher in women (3.58 and 4.34, respectively), while in 2022 and 2023 it was higher in men (3.35 and 3.72, respectively). The greatest difference between sexes occurred in 2023 (3.72 in men vs. 3.17 in women) (Fig. 1). Regarding age groups, the highest hospitalization rate was reported in children under 5 years of age (11.74 per 100,000 inhabitants), followed by a progressive decline reaching a minimum in the 15–19-year age group (2.36). A slight increase was observed in adults aged 65–79 years (3.43) and ≥ 80 years (3.29), with higher values identified at the extremes of age (Fig. 2). The average length of hospital stay for the period was 4.69 days. The highest average was recorded in 2022 (4.90 days), followed by 2021 (4.81 days), while the lowest value was observed in 2024 (4.45 days). Women had a slightly longer average hospital stay than men (4.73 vs. 4.64 days). Overall, the average length of hospital stay showed only minor variations across the study period (Fig. 3).

## 4 Discussion

Salmonellosis remains a relevant enteric infection worldwide and a major cause of morbidity and hospitalization in both industrialized and developing countries (Rosso et al., 2023; Muleta and Meseret, 2025). In the United States, non-typhoidal *Salmonella* infections are estimated to cause approximately 1.35 million illnesses annually, with around 26,500 hospitalizations reported based on national surveillance data (Scallan et al., 2011). Although direct comparisons are limited by differences in surveillance systems and case definitions, the overall hospitalization rate observed in Chile during the 2021–2024 period (3.61 per 100,000 inhabitants) suggests a comparatively lower burden of hospital-treated disease. Despite this, a considerable number of patients in Chile still required hospitalization, with 2672 hospital discharges recorded. These findings reinforce the epidemiological and clinical relevance of non-typhoidal *Salmonella* infections in the Chilean setting.

Regarding age distribution, our findings showed that hospitalization rates were markedly higher among pediatric patients, particularly children under 5 years of age (11.74 per 100,000 inhabitants), which is consistent with previous literature (Galán, 2021; Alfaro-Mora, 2019). Increased susceptibility in this age group has been attributed to immature immune responses, higher fecal-oral exposure, and attendance at childcare facilities (Parra-Payano et al., 2019; Harrison, 2021). Additionally, immunological and microbiota immaturity may further increase vulnerability in young children. In contrast, older adults may be more susceptible due to immunosenescence, comorbidities, and increased exposure to healthcare environments. Similar patterns have been described in other enteric infections and may explain the higher hospitalization rates observed at the extremes of age. Conversely, the lowest hospitalization rate occurred among adolescents aged 15–19 years (2.36 per 100,000 inhabitants). This finding may reflect maturation of the gut microbiota, which during late adolescence becomes predominantly composed of Firmicutes and Bacteroidetes, contributing to improved resistance against enteric pathogens (Björklund et al., 2024). Similarly; the slight increase in hospitalization rates observed among older adults (≥65 years) aligns with the known effects of immunosenescence; increased comorbidity burden; and greater exposure to healthcare settings reported in the literature (Harrison, 2021; Infectologia.info, 2025). Temporal trends during the study period appear to reflect post-pandemic dynamics. The decline observed in 2022 may be related to mobility restrictions, reduced food consumption outside the household, decreased circulation of enteric pathogens, and lower consultation rates for non-respiratory illnesses during the COVID-19 pandemic (Fattinger et al., 2021; Gottlieb et al., 2025). The subsequent increase observed in 2023 and 2024 coincides with the progressive resumption of social activities, reopening of schools and restaurants, and normalization of healthcare services following the pandemic (Pijnacker et al., 2024). Recent reports have described cases of invasive non-typhoidal *Salmonella* infections in patients with COVID-19; potentially related to immune dysregulation and increased susceptibility to secondary bacterial infections during viral illness (Rawson et al., 2020). However; because serotype-level data were not available in the present dataset; this potential relationship could not be evaluated in our study. This phenomenon has been attributed to immune dysregulation and increased susceptibility to secondary bacterial infections during viral illness. However; because serotype-level data were not available in the present dataset; this potential relationship could not be evaluated in our study. Sex-specific analysis showed a slight male predominance in hospitalization rates (3.67 vs. 3.56 per 100;000 inhabitants); although the magnitude of the difference was small. Similar patterns have been reported internationally and may be influenced by biological; hormonal; immunological; or behavioral factors (Nguyen et al., 2022; Guzmán, 2022). Nevertheless, given the limited magnitude of this difference, social and environmental exposures rather than intrinsic biological determinants may better explain this pattern. With respect to clinical outcomes, the average length of hospital stay remained relatively stable throughout the study period, ranging from 4.45 to 4.90 days. Women showed slightly longer hospital stays than men, a trend reported in other bacterial enteric infections and potentially related to differences in healthcare-seeking behavior, comorbidity burden, or clinical management. The reduction in length of stay after 2022 may also reflect the normalization of hospital workflows following COVID-19-related disruptions. In contrast to settings where molecular diagnostics or expanded outpatient management have contributed to shorter hospital stays (Contreras B. et al., 2023; Love et al., 2022), the narrow variability observed in Chile suggests relatively consistent clinical management of non-typhoidal *Salmonella* infections during the study period. Overall, these findings highlight the increased vulnerability of children under 5 years of age and older adults, as well as the post-pandemic rebound in hospital discharges. Given the descriptive nature of the dataset and the limited number of time points available, formal statistical comparisons across years were not performed. Strengthening epidemiological surveillance systems and incorporating serotype-level microbiological data into national reporting systems may improve the monitoring and characterization of severe salmonellosis cases in Chile. Future studies incorporating mortality data would provide a more comprehensive assessment of the severe burden of salmonellosis and should also evaluate the outpatient burden of disease, as well as the role of rapid diagnostics and clinical management strategies on hospitalization trends.

### 4.1 Strengths

• This study provides updated, national-level estimates of hospitalizations due to salmonellosis in Chile during a period of epidemiological and healthcare transition.
• Use of standardized administrative databases (DEIS and INE) ensured consistent case definitions and population denominators across years, allowing for reliable **h**ospitalization rate comparisons over time.
• Stratification by age, sex, and length of hospital stay enabled identification of vulnerable groups and characterization of clinical burden beyond simple case counts.
• The inclusion of post-pandemic years offers valuable context on the recovery of non-respiratory infectious diseases and health service utilization.

### 4.2 Limitations

• Hospital discharge data capture only cases requiring hospitalization and therefore do not reflect the full spectrum of community-managed salmonellosis. However, hospitalization represents a key indicator of severe disease burden and healthcare utilization.
• Serotype-specific information was not available in national databases, preventing differentiation between *S. enteritidis*, *S. typhimurium*, and other serotypes with distinct epidemiological behaviors.
• Administrative data do not provide clinical details such as symptom duration, comorbidities, laboratory confirmation methods, or severity scales, limiting clinical characterization. Additionally, the database provides aggregated annual averages of hospital stay, and measures of variability such as standard deviation could not be calculated.
• The database used in this study contains hospital discharge records but does not include mortality outcomes, preventing the assessment of salmonellosis-related deaths during the study period.
• Incidence calculations rely on population projections, which may not fully account for temporary migration or demographic fluctuations, potentially affecting denominators.
• Outpatient and emergency visits not resulting in hospital admission were not captured. Nevertheless, hospitalizations represent the most clinically severe outcomes and are important indicators of healthcare burden.

## 5 Conclusion

Salmonellosis continues to represent a relevant enteric infection in Chile, generating a non-negligible hospital burden, particularly among children under 5 years of age and older adults. Although the overall hospitalization rate remained low compared to other countries, the post-pandemic increase observed toward 2023–2024 highlights the importance of maintaining active surveillance beyond respiratory pathogens. The stability of hospital length of stay suggests consistent clinical management of non-typhoidal *Salmonella* infections during the study period. To advance prevention and control, it will be necessary to strengthen food safety strategies, improve epidemiological reporting systems, and incorporate serotype-level and outpatient data. These measures would allow for a more comprehensive understanding of severe salmonellosis in Chile and support future public health interventions.

## CRediT authorship contribution statement

**Constanza Pamela Rojas Navia:** Methodology, Investigation, Formal analysis, Conceptualization. **Rocío Belén Herrera Caracciolo:** Software, Project administration, Investigation, Formal analysis, Conceptualization. **Ankiza Nicole Knezevic Altamirano:** Visualization, Validation, Supervision, Investigation, Funding acquisition, Formal analysis, Conceptualization. **Sofía Ignacia Macari Jorquera:** Visualization, Validation, Supervision, Project administration, Methodology, Investigation, Data curation, Conceptualization. **Juan Sanchis-Gimeno:** Resources, Methodology, Funding acquisition, Conceptualization. **Jessica Paola Loaiza Giraldo:** Project administration, Funding acquisition, Conceptualization. **Jose E. León Rojas:** Software, Resources, Project administration, Methodology, Investigation, Formal analysis, Data curation, Conceptualization. **Juan José Valenzuela Fuenzalida:** Software, Resources, Project administration, Methodology, Investigation, Data curation, Conceptualization.

## Ethics approval and consent to participate

Ethical approval was not required because the study used anonymized, publicly accessible secondary data and involved no interaction with human subjects. Therefore, informed consent was not required.

## Funding

This research received no external funding.

## Declaration of competing interest

The authors declare that they have no known competing financial interests or personal relationships that could have appeared to influence the work reported in this paper.

## Data Availability

No data was used for the research described in the article.
